# Cardiac Outpouchings: Definitions, Differential Diagnosis, and Therapeutic Approach

**DOI:** 10.1155/2021/6792643

**Published:** 2021-09-16

**Authors:** Riccardo Scagliola, Gian Marco Rosa, Sara Seitun

**Affiliations:** ^1^Division of Cardiology, Department of Emergency, Cardinal G. Massaia Hospital, Asti, Italy; ^2^Cardiovascular Disease Unit, Department of Internal Medicine, IRCCS Ospedale Policlinico San Martino, University of Genoa, Genoa, Italy; ^3^Department of Radiology and Interventional Radiology, IRCCS Ospedale Policlinico San Martino, Genoa, Italy

## Abstract

**Background and Aims:**

Cardiac outpouchings encounter a series of distinct congenital or acquired entities (i.e. aneurysms, pseudoaneurysms, diverticula, and herniations), whose knowledge is still poorly widespread in clinical practice. This review aims to provide a comprehensive overview focusing on definition, differential diagnosis, and prognostic outcomes of cardiac outpouchings, as well as further insights on therapeutic options, in order to assist physicians in the most appropriate decision-making.

**Methods:**

The material reviewed was obtained by the following search engines: MEDLINE (PubMed), EMBASE, Google Scholar, and Clinical Trials databases, from January 1966 until March 2021. We searched for the following keywords (in title and/or abstract): (“cardiac” OR “heart”) AND (“outpouching” OR “outpouch” OR “aneurysm” OR “pseudoaneurysm” OR “false aneurysm” OR “diverticulum” OR “herniation”). Review articles, original articles, case series, and case reports with literature review were included in our search. Data from patients with congenital or acquired cardiac outpouchings, from prenatal to geriatric age range, were investigated.

**Results:**

Out of the 378 papers initially retrieved, 165 duplicates and 84 records in languages other than English were removed. Among the 129 remaining articles, 76 were included in our research material, on the basis of the following inclusion criteria: (a) papers pertaining to the research topic; (b) peer-reviewed articles; (c) using standardized diagnostic criteria; and (d) reporting raw prevalence data. Location, morphologic features, wall motion abnormalities, and tissue characterization were found to have a significant impact in recognition and differential diagnosis of cardiac outpouchings as well as to play a significant role in defining their natural history and prognostic outcomes.

**Conclusions:**

Careful recognition of cardiac outpouchings remains a diagnostic challenge in clinical practice. Due to a broad cluster of distinctive and heterogeneous entities, their knowledge and timely recognition play a pivotal role in order to provide the most appropriate clinical management and therapeutic approach.

## 1. Introduction

The term “outpouching” generally refers to a physiologic or pathologic evagination of a dilated structure outside the anatomic layer of an organ. In this context, cardiac outpouchings define a broad spectrum of distinctive congenital or acquired entities, whose recognition is of paramount relevance, due to their different natural history, prognostic outcomes, and therapeutic approach [1]. Several parameters, including location, morphology, size, wall motion abnormality, and tissue characterization, deserve careful consideration, in order to provide differential diagnosis for guiding physicians for proper clinical management and therapeutic strategy [2]. Our aim is to provide a comprehensive overview focusing on definition, clinical presentation, natural history and differential diagnosis of cardiac outpouchings, and further findings concerning their prognostic outcomes and therapeutic options.

## 2. Methodology

A narrative review was performed by following the standard methods of the Cochrane Collaboration and PRISMA declaration. The material reviewed was obtained by the following search engines: MEDLINE (PubMed), EMBASE, Google Scholar, and Clinical Trials databases, from January 1966 until March 2021. We searched for the following keywords (in title and/or abstract): (“cardiac” OR “heart”) AND (“outpouching” OR “outpouch” OR “aneurysm” OR “pseudoaneurysm” OR “false aneurysm” OR “diverticulum” OR “herniation”). Review articles, original articles, case series, and case reports with literature review were included in our search. Data from patients with congenital or acquired cardiac outpouchings, from prenatal to geriatric age range, were investigated.

## 3. Results

Out of the 378 papers initially retrieved, 165 duplicates and 84 records in languages other than English were removed. Among the 129 remaining articles, 76 were included in our research material, on the basis of the following inclusion criteria: (a) papers pertaining to the research topic; (b) peer-reviewed articles; (c) using standardized diagnostic criteria; and (d) reporting raw prevalence data.

## 4. Prevalence and Types

### 4.1. Cardiac Aneurysms

Cardiac aneurysms are defined as outpouchings involving endocardium, epicardium, and thinned, scarred myocardium, which exhibit regional wall motion abnormalities (more frequently, dyskinesia) with paradoxical bulging expansion during systole. Histopathological findings often detect fibrous connective tissue interposed to spared muscular fibers, with scarring tissue and mononuclear cells [3]. Distinctive morphological features for true cardiac aneurysms on imaging tools generally include a laminar blood flow through a wide neck, a smooth transition from normal myocardium to thinned aneurysmal wall, and a ratio of the maximum neck diameter to the maximum aneurysm diameter greater than 0.5 (usually 0.9 to 1.0) [4]. Although true aneurysms can be detected in all the cardiac chambers, most frequently they involve the left ventricle, particularly as a consequence of transmural myocardial infarctions. In this clinical setting, previous studies in the thrombolytic era showed a significant decreased incidence in patients exhibiting a patent infarct-related artery than those with non-revascularized coronary artery disease (from 18.8% to 7.2%) [5]. Data from the contemporary percutaneous coronary intervention era showed a further decline in the absolute incidence of left ventricular (LV) aneurysms in ischemic subjects (ranging from 5% to 15%, on the basis of epidemiologic data reported in the literature), due to the major advances and improved techniques of coronary revascularization. However, the residual detected cases are still related to a detrimental clinical outcome in the long-term follow-up [6, 7]. Besides ischemic heart disease, which still represents the most prevalent cause for acquired aneurysms, nonischemic aetiologies are described in less than 5% of total cases [6]. Among them, chest trauma, cardiac surgery, and hypertrophic cardiomyopathy (HCM) are the mostly associated conditions, while Chagas disease, mucopolysaccharidosis, and sarcoidosis are other less frequently reported causes [8–12]. Finally, up to one-third of patients with nonischemic aneurysms have no identifiable aetiology and are classified as idiopathic [13]. More frequently, acquired LV aneurysms involve cardiac apex or anterior wall, usually arising from the occlusion of the anterior descending coronary artery, while only 3% of them affect the LV posterior wall. A clinical preponderance for apical or anterior locations can be related to the high lethal rate of extensive posterior myocardial infarctions, which often involve the posteromedial papillary muscle, usually resulting in severe mitral regurgitation so that such patients are more frequently prone to die over the time, rather than develop posterior LV aneurysm. Furthermore, the more posterior is true aneurysm, and the more difficult is its detection by usual imaging modalities, resulting in a diagnostic bias in favour of anterior or apical aneurysms [14, 15]. LV aneurysms may be rarely discovered in childhood, with an overall incidence raising 0.34% [16]. Their aetiology is supposed to be acquired during the prenatal period and include intrauterine viral infections, coronary hypoplasia, stenosis, or intimal proliferation [17, 18]. More rarely, cardiac aneurysms can be detected in the right ventricular (RV) chamber or right ventricular outflow tract (RVOT). Most of them are congenital and have been described to be associated with other cardiac abnormalities, such as the atrialized ventricular portion in Ebstein anomaly, pericardial agenesis, Uhl anomaly, and arrhythmogenic right ventricular dysplasia (ARVD) [19]. In the latter case, the presence of focal localized aneurysms is described as multiple areas of outward dyskinetic bulges in the RV free wall, which persist in systole and diastole. Regional RV aneurysms are currently included in the echocardiographic findings of the contemporary Task Force Criteria of ARVD (together with RV wall motion abnormalities, dilated RVOT, and reduced fractional area change) and must not be confused with the “accordion sign” (defined as a subtle crinkling of the RVOT or the subtricuspid region of the RV free wall, becoming more prominent during systole), which can be detected in ARVD subjects [20, 21]. Such structural abnormalities actively contribute to the degree of RV impairment in patient with ARVD. However, despite limited data are reported in the literature, to date, the degree and amount of the underlying myocardial fibro-adipose replacement still represent the strongest predictor of arrhythmogenic risk in this subset population [22]. Posterior mitral annulus is another uncommon location of nonischemic aneurysms, often arising from a congenital disjunction between LV myocardium and perimitral left atrial region, which predisposes to a weakened myocardial area and development of submitral aneurysms [23]. However, reports from the literature also described other potential causes, such as trauma, tuberculosis, human immunodeficiency virus infection, rheumatic carditis, and Takayasu disease, suggesting an acquired aetiology [24]. Finally, even though atrial chambers may be rarely involved by true aneurysms, right atrial aneurysms are more often congenital and are rarely detected during the intrauterine life. They may be associated with other congenital heart diseases, particularly with atrial or ventricular septal defects [25]. Left atrial aneurysms mostly involve the left atrial appendage, and as opposed to right atrial aneurysms, they are more commonly related to acquired pathologic processes (including rheumatic heart disease, syphilitic, or tubercular myocarditis) [26]. Clinical findings widely range from totally asymptomatic subjects, to patients presenting nonspecific symptoms, such as exertional dyspnoea, chest pain, or hypotension, until clinical complications, including heart failure, systemic thromboembolism, and ventricular arrhythmias [15, 27]. However, unlike cardiac pseudoaneurysms, true aneurysms carry a low risk of rupture; therefore, differentiation between these two entities is crucial in order to provide the most appropriate therapeutic strategy [28].

### 4.2. Cardiac Pseudoaneurysms

As opposed to true aneurysms, cardiac pseudoaneurysms (also known as “false aneurysms”) are defined as outpouchings created when cardiac rupture is contained by overlying adherent pericardium and thrombotic material, with no myocardial tissue, thus preventing self-evident cardiac tamponade and death [2, 29]. The available data from case reports and case series from the literature showed a prevalence of cardiac pseudoaneurysms following transmural myocardial infarctions, ranging from 0.2% to 0.3%, mostly after wide ischemic heart disease involving male and elderly patients [30]. Less commonly, cardiac pseudoaneurysms occur after other clinical conditions, such as cardiac surgery (mainly mitral or aortic valve replacement), chest trauma, and infections, as reported in the systematic review of 290 cases by Frances and colleagues [31]. Morphologically, the presence of a narrow neck connecting a globular, echo-free cavity to the cardiac chamber, an abrupt transition from normal myocardium to the outpouching, a turbulent flow through the aneurismal neck, and a maximum orifice to chamber diameter ratio less than 0.5 (generally 0.25 to 0.5) are the main distinctive features of a pseudoaneurysm. Additionally, a recent analysis has reported that a decrease greater than 50% of the outpouching wall thickness measured at 1 cm from the aneurysmal neck would be considered a new sensitive and specific marker for diagnosing false aneurysms [32, 33]. As for true aneurysms, the most frequent location for cardiac pseudoaneurysms is the left ventricle. However, as opposed to true aneurysms, posterior LV pseudoaneurysms are more than twice as common as false aneurysms involving other ventricular regions and mostly result after myocardial infarctions following circumflex coronary artery occlusion [34]. One proposed explanation comes to the fact that anterior wall rupture of the left ventricle may be more likely prone to hemopericardium and immediate death than posterior LV rupture. By the contrary, because of the recumbent decubitus of hospitalized patients, an inflammatory reaction involving the posterior pericardium may more often result in cardiac rupture and left pseudoaneurysm formation, rather than cardiac tamponade [31, 35]. More rarely, cardiac pseudoaneurysms involve the right ventricle, mainly following iatrogenic procedures (postcardiac surgery, device lead extraction, and endomyocardial biopsy) or consequent to penetrating chest wall trauma [36]. Other potential locations include the mitral-aortic intervalvular fibrosa (mostly due to infectious processes or following valvular surgery) and cardiac atria (rarely related to ablative procedures and more often subsequent to blunt chest trauma) [37, 38]. Specifically, right atrium is the cardiac chamber mostly affected in blunt chest injuries and is involved in nearly 40% of total cases of cardiac trauma, due to its thin wall and anterior location than other cardiac structures [39]. Clinical presentation of cardiac pseudoaneurysms is extremely heterogeneous: a case series reported by Yeo et al. showed a percentage of patients completely asymptomatic lying in between 10% and 48% [40]. Most commonly, symptoms are usually nonspecific and often indistinguishable for true aneurysms, while other clinical presentations, such as sudden death, congestive heart failure, systemic thromboembolism, and ventricular arrhythmias, are less frequently reported. Clinical signs may include an apical impulse (in case of particularly extensive pseudoaneurysms) or a third sound in case of interference between the false aneurysm and LV filling, while a to-and-fro murmur can be the result of blood flow through the pseudoaneurysmal sac, as well as consequent to associate mitral regurgitation [41, 42]. As opposed to true aneurysms, cardiac pseudoaneurysms still carry a higher early risk of rupture, lacking the structural support of the myocardial layer. So, a deepening consideration about surgical treatment should be closely taken into account [2, 43].

### 4.3. Cardiac Diverticula

Cardiac diverticula are described as congenital outpouchings which contain all three layers of cardiac wall (endocardium, myocardium, and pericardium) and display a synchronous contractility with the corresponding cardiac chamber, as compared to aneurysms and pseudoaneurysms (which are akinetic or dyskinetic outpouchings) [1, 28]. Although no recent reports have been explained, the current epidemiology of cardiac diverticula and early studies showed a prevalence rate of 0.4% among 750 cardiac necropsy cases, while one analysis by multidetector computed tomography reported an increased prevalence to 2.2% [44]. Their pathogenic process has been postulated to be the result of failure in the fusion of the cardiac loop to the yolk sac during the fourth embryonic week. These structures are then stretched out during the subsequent phases of embryological development. Intrauterine viral infections, muscular or connective tissue defects, and excessive primordial cell stimulation have been considered as plausible favouring conditions, leading to a disrupted embryogenic process [45]. Diverticula can be observed in all the cardiac chambers: they involve mostly the left ventricle and in a less percentage the right ventricle or both the ventricular chambers (with a numerical ratio of almost 8 : 1 : 1) [46]. LV diverticula are classified as apical or nonapical, on the basis of their location: apical diverticula are more prevalent and described as finger-like or hook-like contractile pouches, typically less than 3 cm in length and 1.25 cm in width, with a narrow connection to the ventricular chamber [47]. In more than 70% of cases, they are described as part of a syndrome firstly described by Cantrell et al. in 1953, characterized by midline thoracoabdominal defects (including omphalocele, epigastria hernia, diaphragmatic rents, and sternal abnormalities), congenital heart disease (more often tetralogy of Fallot, ventricular septal defects, atrial septal defects, and tricuspid atresia), mesocardia or dextrocardia, and partial absence of diaphragmatic pericardium [48]. The most frequent location of LV diverticula at the cardiac apex and their reported association with abdominal wall defects would be referred to the failure in differentiating the primitive mesoderm into its splanchnic and somatic layers since the 14^th^ day of embryonic life [46]. On the other hand, nonapical diverticula are generally described as isolated cardiac defects of multiple shapes and sizes (ranging from 0.5 cm to 9 cm), with a narrow or wide connection to the cardiac chamber, usually arising from the LV anterior wall or subaortic region, and more rarely involving the RV chamber or both ventricles [47, 49]. Cardiac diverticula are also rarely recognized in the atrial chambers, especially in the left atrium. They are commonly located in the anterosuperior wall and have generally a broad neck and a smooth surface. Atrial diverticula should not be confounded with accessory atrial appendages, which are mostly located in the lateral and inferior walls (along the line of fusion between pulmonary veins and the embryonic atrial chamber), usually present a narrow neck and an irregular surface, due to the presence of pectinate muscles [1]. Cardiac diverticula are often asymptomatic and can be incidentally detected by imaging techniques. Their sizes generally do not change over the time, suggesting a benign course, and in few cases, spontaneous regression has been noticed. For these reasons, although clear recommendations are still very difficult to derive, a close follow-up and monitoring is often pursued, reserving further management only for symptomatic patients, and in case of potential complications (like thromboembolism or conduction disturbances) or associated abnormalities [2, 50].

### 4.4. Cardiac Herniations

Cardiac herniations are rare and life-threatening findings, characterized by a myocardial protrusion through a pericardial tear. Their aetiology includes congenital defects, chest trauma, or iatrogenic (postsurgical) procedures [51]. Congenital pericardial defects are generally uncommon findings, whose prevalence ranges from 0.002% to 0.004%, in accordance with surgical and histopathological series reported in the literature [52]. They occur from the premature atrophy of the common cardinal vein and vary from partial pericardial agenesis (more frequently involving the left hemipericardium) to the complete absence of the entire pericardium. Albeit often described as isolated findings, they may be associated with other congenital cardiac abnormalities, including atrial septal defects, bicuspid aortic valve, patent ductus arteriosus, or pulmonary malformations [53]. On the other side, traumatic pericardial tears represent another leading cause of cardiac herniations: they may follow blunt or penetrating injuries, which may be responsible for pericardial damage due to the compression between the sternum and vertebral column or to a direct traumatic injury, respectively [54]. Most of the patients develop pericardial tears from blunt chest trauma: their prevalence ranges from 0.5% to 1% of case series reported in the literature. Pericardial defects longer than 8–12 cm in length are more prone to be associated with cardiac herniations, which are almost confined to the left side, while they are rarely subsequent to diaphragmatic pericardial tears (encountering fewer than 20% of total cases) [55, 56]. Finally, cardiac herniations have been described as iatrogenic findings following surgical procedures, including pneumonectomy or lobectomy with associated pericardiotomy or pericardiectomy, as well as uncommon complications during cardiac surgery [57, 58]. Clinical presentation is somewhat variable, from asymptomatic subjects in case of short pericardial defects to cardiac displacement leading to pleural effusions, atelectasis, tension pneumothorax, or pneumopericardium until myocardial strangulation and even sudden cardiac death. Most frequently, in cases of right-sided pericardial rupture, cardiac herniation into the right thorax may bring to deranging and even fatal hemodynamic consequences, due to the distortion of atriocaval junctions and great vessels, with consequent reduction in venous return and critical fall of cardiac output. In this acute clinical setting, emergency thoracotomy seems to be the only effective resuscitating strategy [56, 59].

### 4.5. Myocardial Crypts and Recesses

Unlike cardiac diverticula, which are outpouchings extending beyond the borders of myocardial wall, myocardial crypts (sometimes interchangeably named as “clefts” or “crevices”) are defined as congenital narrow, deep blood-filled invaginations confined to the otherwise compacted myocardium, and characterized by a penetration rate of more than 50% of myocardial thickness in diastole and a subtotal or total obliteration during systole. They differ from myocardial recesses (even defined as “partial crypts”), which are defined as physiologic irregularities of ventricular wall, involving less than 50% of myocardial thickness [60]. Both of these structural findings develop perpendicularly to the endocardial border of the ventricular myocardium and do not extend beyond the epicardial boundary. For these reasons, such entities cannot be essentially encountered as cardiac outpouchings, although they may mimic their appearance, due to their morphological features [1]. Progressive advances in imaging techniques have improved the identification of myocardial crypts and recesses, with an overall prevalence of 6-7% in general population [61]. Specifically, data published by Maron and colleagues remarked a strong relationship between myocardial crypts and HCM patients, especially in those with a phenotype-negative pattern [62]. These findings were confirmed by the analysis of Brouwer et al., who raised the hypothesis that these defects could be used as early predictors of gene carrier status in HCM [63]. In the same line, Germans and coworkers reported the detection of myocardial crypts by cardiac magnetic resonance in the inferoseptal region of a significant percentage of patients with mutations for HCM, although they had not developed ventricular hypertrophy yet [64]. Finally, the analysis by Johansson et al. involving a long series of 399 subjects, revealed the presence of myocardial crypts in two different locations: at the level of the inferior basal segment and at the mid to apical septal level, both in HCM and hypertensive patients, as well as in subjects with surgically repaired congenital heart disease and in healthy volunteers [65]. Despite their etiopathogenesis remains unclear, both crypts and recesses seem to share a common origin, related to a defective reabsorption of ventricular myocardial trabeculations during the embryological period. In this regard, Singh et al. have supposed a common developmental anomaly shared with isolated ventricular noncompaction, raising the hypothesis of being part of a single continuum disease [66]. However, several features classically differentiate ventricular noncompaction from the aforementioned structural entities, including (a) the presence of a two-layered myocardium with a thin compacted epicardial band and a thicker, noncompacted endocardial border; (b) a maximal end-systolic ratio between noncompacted and compacted myocardial layers greater than 2; (c) the evidence of more than three deeply perfused communicating intertrabecular recesses with colour Doppler echocardiography, and (d) the frequent detection of clinical and subclinical systolic impairment of the affected myocardial segments [67, 68]. Other potential aetiologies include myocardial ischemia from microvascular dysfunction, and myocardial disarray, histologically characterized by crossing and interdigitation of cardiomyocytes [64, 69]. Natural history of myocardial crypts and recesses is unremarkable, and to date, no evidence of links with pathologic consequences has been reported. Therefore, these imaging findings deserve recognition and have to be distinguished from other confounding entities, due to their different prognostic outcome.

## 5. Imaging Diagnostic Techniques

### 5.1. Chest Radiography

Alterations of cardiac silhouette can be underscored on chest radiography, on the basis of the size and location of cardiac outpouchings. They may range from an unusual prominent contour of the heart (“boot shaped”), until a marked protrusion of the inferior left cardiac dome (mostly observed in patients with extensive true LV aneurysms or pseudoaneurysms) and cardiomegaly. Calcifications to the boundaries of aneurysm and pseudoaneurysm sac could be detected in the later stages. Other radiological findings may include the apparent elevation of the cardiac apex, a prominent contour of pulmonary artery, and rightward or leftward cardiac displacement and rotation, sometimes associated with pleural effusions, atelectasis, or tension pneumothorax (especially in the case of huge cardiac herniations associated with broad pericardial tears) [46, 70].

### 5.2. Echocardiography

Transthoracic and transoesophageal echocardiography, particularly with contrast enhancement, are widely available noninvasive imaging modalities, which usually represent the reasonable first-line tool for clarifying the diagnosis of cardiac outpouchings. However, they are often unable in confirming a definitive instrumental diagnosis (reaching a diagnostic accuracy of 26% and 75%, respectively), due to the lower spatial resolution than other imaging techniques [41, 46]. Tissue-Doppler and strain and strain rate echocardiography provide further insights in detecting lesions with normal, absent, or paradoxical regional contractility, while contrast harmonic power Doppler provides additional findings on kinesis and further insights on myocardial vascular perfusion. Furthermore, 3D-echocardiographic reconstruction allows to calculate the volume of cardiac outpouchings [71–73]. Nonconventional views, including off-axis projections, are often necessary to identify even small continuum solutions in the myocardial wall and echo-free spaces outside the cardiac chambers. Moreover, also the identification of mural thrombi and their echocardiographic features deserve further attention because blood-pool imaging may underestimate the maximum neck or echo-free cavity diameters, if they are lined with thrombotic material [42, 74]. Additionally, Doppler techniques may give further information in identifying the nature of blood flow through the orifice or within the aneurysmal chamber. The presence of turbulent flow through the neck of the cavity, detected by pulsed wave and colour Doppler, suggests the presence of a pseudoaneurysm, while a laminar flow usually predisposes for true aneurysm. Bidirectional colour-flow Doppler between an extracardiac echo-free space and the ventricular chamber also permits to distinguish between simple pericardial effusion and ventricular outpouching [75]. Echocardiography has also been described as a useful tool in recognizing short pericardial tears and cardiac herniations, although data from the literature have reported a low sensitivity for detecting longer pericardial defects [70].

### 5.3. Radionuclide Techniques

Although the use of radionuclide techniques using perfusion markers for the evaluation of myocardial viability has been reported from over 20 years ago, the experience with this modality in such context is poor. Several perfusion markers, such as technetium- (^99m^Tc-) tetrofosmin and iodine- (^123^I-)*β*-methyl-iodophenyl-pentadecanoic acid, and mostly, ^18^F-fluorodeoxyglucose, are used to detect the active myocardium, on the basis of their reuptake from the cardiac wall. Furthermore, they can be helpful in assessing the risk of rupture of the cardiac outpouching. However, to date, the use of these techniques is still limited, due to the low spatial resolution and the availability of alternative diagnostic tools, which provide a better anatomic definition and tissue characterization [42, 46].

### 5.4. Chest Computed Tomography

Only few data reported in the literature systematically analysed the role of chest computed tomography (CT) in the diagnosis of ventricular outpouchings. Multislice CT has been shown to provide a three-dimensional reconstruction set, allowing further geometrical characterization of cardiac outpouchings, as well as a greater sensitivity in identifying changes of cardiac axis and pericardial discontinuity [56, 76]. Cardiac CT also provides further findings on coronary anatomy, albeit limited by the increased heart rate (greater than 70 beats per min). Taken together, these findings may play a relevant role in therapeutic decision-making [77].

### 5.5. Magnetic Resonance Imaging

Magnetic resonance imaging (MRI) allows an excellent tissue characterization, providing a clear distinction between pericardium, myocardium, and thrombotic stratifications. Viable images with late gadolinium enhancement accurately show fibrous, scarring myocardial areas and strongly correlate with the improvement of contractility rates following myocardial revascularization, while cine-MRI permit a better identification of regional wall motion abnormalities. Furthermore, MRI plays a crucial role in differentiating cardiac outpouchings from other underlying cardiac anomalies, including congenital heart disease or ARVD [2]. For the aforementioned reasons, cardiac MRI has become the gold standard imaging technique for the detection and differential diagnosis of cardiac outpouchings, with a reported sensitivity of 100% and specificity of 83% [78]. Cardiac diverticula usually show a synchronous contractility on cine-MRI, together with the absence of enhancement in the myocardial wall and overlying pericardium, while true ventricular aneurysms are generally characterized by a dyskinetic, delayed enhanced wall without pericardial enhancement, referring to thinned, scarred myocardium. Fatty replacement of scarred myocardial wall, parietal calcifications, and thrombotic material can be detected in chronic aneurysms. Conversely, pseudoaneurysms are often identified by the obliteration of the myocardial-pericardial interface and sharp, delayed enhancement of overlying pericardium, which has been hypothesized as a consequence of pericardial chemical irritation by blood released during the acute phase of cardiac rupture, leading to an inflammatory reaction and pericardial neovascularization [2, 79]. Cardiac MRI can also be used in detecting pericardial defects and cardiac herniations. However, such identifications are not always straightforward because their appearance is often intermittent in time and varies with the patient positional changes, as well as because pericardial layers are often difficult to depict over the left-sided chambers (where pericardial defects are most frequently located), due to the paucity of surrounding fat [80]. The main characterizing features of cardiac outpouchings are summarized in Table 1.

### 5.6. Cardiac Catheterization

Albeit rarely used, due to the invasive nature of the technique and the use of ionizing radiations, cardiac catheterization provides further insights which help to characterize cardiac outpouchings, including changes in myocardial contractility, the sizing of the neck connecting the cavity to the cardiac chamber, the prolonged staying of contrast medium, and the presence or absence of surrounding coronary arteries [81]. Furthermore, according to the observation firstly postulated by Spindola-Franco and Kronacher, coronary angiography may help in identifying the true location of the epicardium, which can be useful for differentiating true ventricular aneurysms (that are outpouchings bounded by epicardium, which do not extend beyond coronary arteries) from pseudoaneurysms (whose cavity lies outside the epicardium and hence cannot be “draped over” by coronary arteries) [82]. Additionally, coronary angiography is usually required before surgical procedures, in order to assess the need for concomitant bypass grafting, on the basis of the degree and extension of coronary artery disease. Based on the published reported data, LV angiography has been reported to reach a definitive differential diagnosis between true aneurysms and pseudoaneurysms for more than 85% of patients, while a less percentage of cases were missed when X-ray tube was not perpendicular to the outpouching (thus resulting in a partial overlap with the left ventricle) or in the cases of inadequate use of contrast medium [31].

## 6. Prognostic Outcome and Treatment

**Figure 1:**
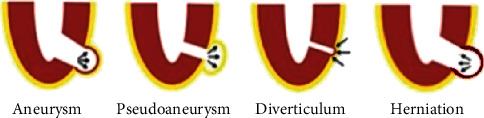
Summary illustration including each type of cardiac outpouching (adapted from A. Cresti et al., J Cardiovasc Echography, 28, 9–17. 2018).

Therapeutic decision-making for cardiac outpouching is often challenging, due to a broad and heterogeneous cluster of clinical entities, each with a different natural course and prognostic outcome. Untreated ventricular pseudoaneurysms carry a considerable risk of rupture, ranging from 30% to 45% (mainly within the first months after an acute coronary syndrome), due to the lack of myocardial wall [41, 43]. So, an urgent surgical treatment is often required, compared to true aneurysms, which are less prone to rupture and are often treated medically, reserving surgical repair only for patients with associated complications, including recurrent heart failure, systemic embolism, or refractory arrhythmias [42, 83]. Active surgical management with primary closure or patch plasty is mandatory for patients with wide and symptomatic pseudoaneurysms, as well as in acute clinical settings. On the other side, several reports have recently advocated a conservative management for asymptomatic patients, especially for those with chronic pseudoaneurysms (defined as lasting longer than 3 months), with lesion size less than 3 cm, incidentally found on imaging techniques and with high surgical risk [29, 84]. However, although few data from the literature did not show an increased mortality risk for patients treated with medical therapy alone, other reports underlined a higher risk of rupture to 48%, described for patients with asymptomatic chronic pseudoaneurysms, who did not undergo surgical repair [85]. Therefore, critical decision-making on the most appropriate therapeutic strategy, in light with the benefit and detriments of surgical approach, is warranted. Percutaneous device closure of ventricular pseudoaneurysms remains an alternative of choice, particularly for subjects with smaller lesions, high surgical risk, and in those requiring a redo cardiac surgery [86]. A deep argumentation is also deserved for the management of cardiac diverticula, for which there are no clear recommendations yet, due to the lack of extensive case series in the literature, and the absence of specific guidelines on therapeutic options. Therefore, decision-making is generally based on the patient's clinical findings, the size and extension of the diverticulum, and the presence of other associated defects [46]. Surgical treatment is usually recommended in symptomatic patients and/or with concomitant congenital anomalies: in the majority of cases, in which diverticular neck is less than 2 cm in size, surgical repair by direct suture of the orifice is performed, while for diverticular neck larger than 2 cm, surgical resection and closure with pericardial, Dacron, or polytetrafluoroethylene patch is performed [87, 88]. Conversely, management of patients with asymptomatic and isolated cardiac diverticula remains an unresolved concern. Although some authors have previously proposed systematic surgical treatment in order to prevent potential adverse complications (including embolism, arrhythmias, or heart failure), a conservative strategy with close follow-up in the latter cases has gradually taken shape, due to the relatively good long-term outcome and the risk of postsurgical complications [49, 89, 90]. Finally, albeit uncommon, cardiac herniations through pericardial tears are graved by a high mortality rate, lying in between 30% and 64%, especially for symptomatic patients with longer pericardial defects, and in critical care scenarios [91]. Right-sided cardiac herniations are prone to detrimental hemodynamic consequences, caused by the twisting of great vessels, with consequent impaired venous outflow and reduced cardiac output. Therefore, for herniations renting right-sided pericardial defects, surgical repair of pericardial tear with primary direct suture or by patch plasty with synthetic or autologous material (usually parietal pleura or fascia lata) is warranted. Conversely, left-sided herniations predispose to a less hemodynamic impairment, although they can bring to myocardial strangulation, resulting in ischemic injury and potential lethal conduction disturbances, like ventricular arrhythmias. So, in the latter cases, an alternative therapeutic solution to surgical closure of pericardial tear is represented by the enlargement of left-sided defect, in order to prevent cardiac strangulation, while the same approach does not prevent hemodynamic consequences of cardiac dislocation through right-sided pericardial defects [59, 92]. Taken together, such recommendations underline the need of an individualized therapeutic strategy, based on the patient's clinical profile, the type and features of cardiac outpouching, clinical comorbidities, and the likelihood of adverse events or complications. A summary illustration including each type of cardiac outpouching has been shown in Figure 1.

## 7. Conclusions

In conclusion, careful recognition of cardiac outpouchings remains a diagnostic challenge in clinical practice. Because of a broad cluster of distinctive entities, either by morphological features and prognostic outcomes, their knowledge and timely characterization by imaging tools play a pivotal role in order to provide the most appropriate clinical management and therapeutic approach.

## Figures and Tables

**Table 1:** Characterizing features of cardiac outpouchings.

Cardiac outpouching features	Aneurysm	Pseudoaneurysm	Diverticulum	Herniation
Common anatomic location	Cardiac apex; LV anterior wall	LV posterior wall	Cardiac apex (apical) LV anterior wall; subaortic (nonapical)	Variable
Neck	Wide	Narrow	Variable	Variable
Neck/chamber ratio	0.9–1.0	0.25–0.5	Variable	Variable
Contractility	Dyskinesia	Akinesia	Synchronous	Synchronous
MRI appearance	Delayed enhancement of the myocardial wall	Delayed enhancement of the overlying pericardium	Normal myocardial and pericardial signal intensity	Normal myocardial signal intensity and discontinuity of overlying pericardium
Histology	Fibrous tissue interposed to spared myocardium	Pericardium with mural thrombus	Endocardium, myocardium, and pericardium	Endocardium and myocardium

LV: left ventricle; MRI: magnetic resonance imaging.
